# Genetic underpinnings explored: *OPA1* deletion and complex phenotypes on chromosome 3q29

**DOI:** 10.1186/s12920-024-01850-6

**Published:** 2024-04-19

**Authors:** Ethan Hung-Hsi Wang, Pei-Hsuan Lin, Pei-Liang Wu, Eugene Yu-Chuan Kang, Laura Liu, Lung-Kun Yeh, Kuan-Jen Chen, Meng-Chang Hsiao, Nan-Kai Wang

**Affiliations:** 1 Department of Ophthalmology, Edward S. Harkness Eye Institute, Columbia University, New York, NY USA; 2 College of Arts and Sciences, University of Miami, Coral Gables, FL USA; 3 Department of Ophthalmology, National Taiwan University Yunlin Branch, Yunlin, Taiwan; 4 College of Medicine, National Taiwan University, Taipei, Taiwan; 5 College of Medicine, Chang Gung University, Taoyuan, Taiwan; 6 Department of Ophthalmology, Linkou Medical Center, Chang Gung Memorial Hospital, Taoyuan, Taiwan; 7 Graduate Institute of Clinical Medical Sciences, College of Medicine, Chang Gung University, Taoyuan, Taiwan; 8 Department of Pathology, Microbiology and Immunology, Vanderbilt University Medical Center, Nashville, USA; 9 School of Traditional Chinese Medicine, Chang Gung University, Taoyuan, Taiwan; 10 Vagelos College of Physicians and Surgeons, Columbia University, New York, USA; 11 Edward S. Harkness Eye Institute, Columbia University Irving Medical Center, Hammer Health Sciences Building, 701 W. 168th St, 10032 New York, NY USA

**Keywords:** Autosomal dominant optic atrophy, Brain aneurysm, *OPA1*, *Exome sequencing*, *Microarray*

## Abstract

**Background:**

Copy number variations (CNVs) have emerged as significant contributors to the elusive genetic causality of inherited eye diseases. In this study, we describe a case with optic atrophy and a brain aneurysm, in which a *de novo* CNV 3q29 deletion was identified.

**Case presentation:**

A 40-year-old female patient was referred to our department after undergoing aneurysm transcatheter arterial embolization for a brain aneurysm. She had no history of systemic diseases, except for unsatisfactory best-corrected visual acuity (BCVA) since elementary school. Electrophysiological tests confirmed the findings in retinal images, indicating optic nerve atrophy. Chromosomal microarray analysis revealed a *de novo* deletion spanning 960 kb on chromosome 3q29, encompassing *OPA1* and six neighboring genes. Unlike previously reported deletions in this region associated with optic atrophy, neuropsychiatric disorders, and obesity, this patient displayed a unique combination of optic atrophy and a brain aneurysm. However, there is no causal relationship between the brain aneurysm and the CNV.

**Conclusion:**

In conclusion, the optic atrophy is conclusively attributed to the OPA1 deletion, and the aneurysm could be a coincidental association. The report emphasizes the likelihood of underestimating *OPA1* deletions due to sequencing technology limitations. Recognizing these constraints, healthcare professionals must acknowledge these limitations and consistently search for *OPA1* variants/deletions in Autosomal Dominant Optic Atrophy (ADOA) patients with negative sequencing results. This strategic approach ensures a more comprehensive exploration of copy-number variations, ultimately enhancing diagnostic precision in the field of genetic disorders.

**Supplementary Information:**

The online version contains supplementary material available at 10.1186/s12920-024-01850-6.

## Background

The nuclear *OPA1* gene encodes for a dynamin-like GTPhase protein (OPA1 Protein) located in the inner mitochondrial space, responsible for mitochondrial dynamics (inner membrane fusion), cristal integrity, energetics, and mitochondrial DNA (mtDNA) maintenance. Variants in the *OPA1* gene will disrupt mitochondrial functions, causing a mitochondrial disease known as Autosomal Dominant Optic Atrophy (ADOA). ADOA is characterized by the selective degeneration of retinal ganglion cells (RGC), which leads to progressive visual loss. Up to 20% of *OPA1* variant carriers reported having ADOA plus, which includes extra ocular clinical features of myopathy, peripheral neuropathy, ataxia, encephalopathy, sensorineural hearing loss, and chronic progressive external ophthalmoplegia [1].

Exome Sequencing is considered cost-effective compared to Genome Sequencing because it specifically targets the protein-coding regions of the genome, known as the exome. Exome Sequencing is a valuable tool for identifying disease-causing variants in genetic disorders, especially when sequencing specific regions of interest. The techniques used in Exome Sequencing are capable of detecting low-level genetic variants that may be missed by traditional Sanger sequencing [2]. However, Exome Sequencing has its limitations. It does not cover variants in non-coding regions of the genome. In such cases, Sanger sequencing or Genome Sequencing may be more suitable alternatives. Moreover, some genes may have inadequate coverage in Exome Sequencing due to highly repetitive sequences, such as those found in the open-reading frame 15 (ORF15) region of the *RPGR* gene or in mitochondrial DNA (mtDNA). In these instances, Sanger sequencing or targeted panels for mtDNA are more advantageous. Exome Sequencing also falls short in identifying structural variations like deletions, duplications, translocations, and inversions. It is worth noting that copy number variation (CNV), a common genetic variation in the human genome [3], has recently emerged as a significant contributor to the genetic basis of inherited eye diseases [4].

In this case, we report a *de novo* 960 kb deletion in 3q29 that was presented with both optic atrophy and a brain aneurysm. Within this deletion, the haploinsufficiency of *OPA1*, associated with autosomal dominant optic atrophy, is likely responsible for the ophthalmological anomalies. The presence of the aneurysm could be coincidental.

### Case presentation

**Fig. 1:**
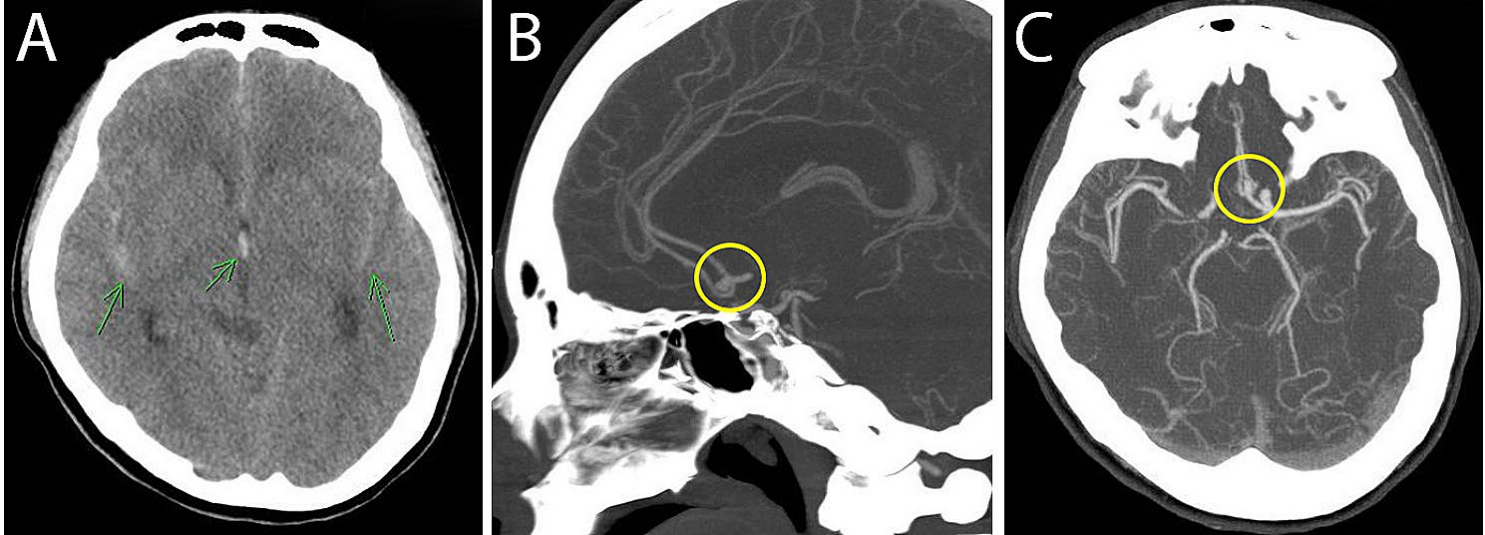
Brain computed tomography (CT). **(A)** Brain CT revealed a diffuse subarachnoid hemorrhage (green arrows). **(B&C)** The CT angiography also showed a small aneurysm with lobulated contour, 4.6 mm in dimension noted in anterior communicating artery (yellow circles**)**

A 40-year-old female without previous medical history was brought to the emergency room at Chang Gung Memorial Hospital, Taiwan, after suffering from severe headache and vomiting. Brain computed tomography (CT) revealed a diffuse subarachnoid hemorrhage (SAH) with a hematoma measuring one mm, diffuse brain swelling and hydrocephalus (green arrows Fig. 1A). The CT angiography also showed a small aneurysm with lobulated contour, 4.6 mm in dimension noted in anterior communicating artery (yellow circles Fig. 1B and C). Aneurysm transcatheter arterial embolization was performed following the discovery. The patient was then reported to the Department of Ophthalmology for postoperative examination.

**Fig. 2:**
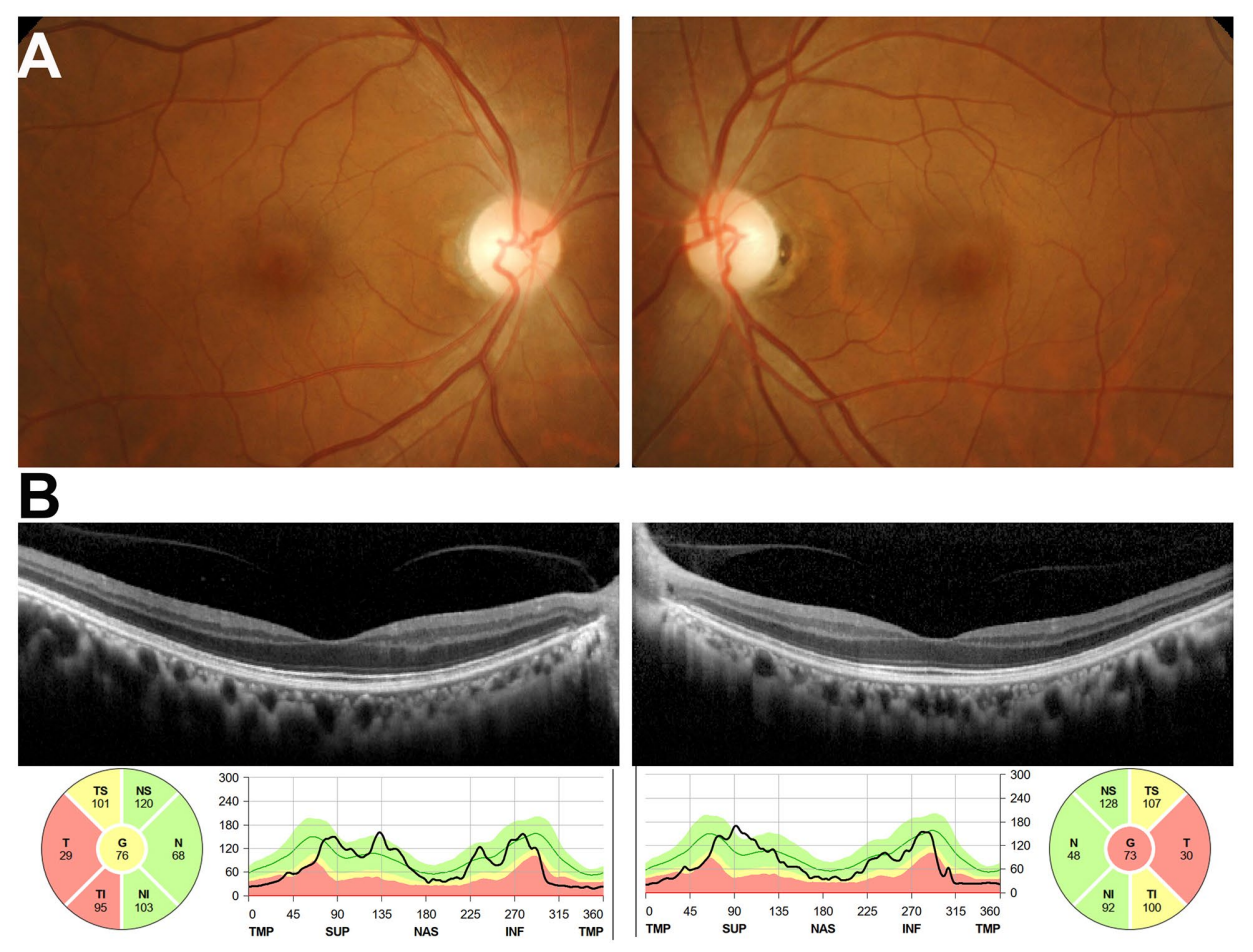
Retinal images. **(A)** Fundus photo showed temporal optic nerve pallor. **(B)** The spectral-domain optical coherence tomography revealed a normal retina structure, with thinning of the retina nerve fiber layer around the optic nerve head, depicting optic atrophy

**Fig. 3:**
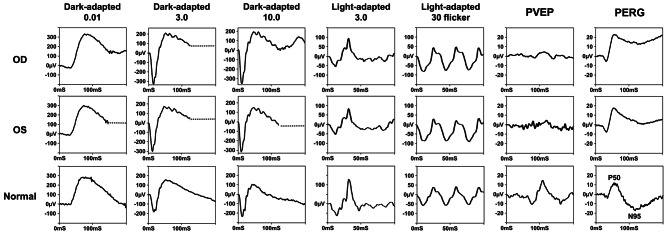
Ocular electrophysiology. The full field electroretinogram (ERG) showed normal scotopic and photopic responses. The pattern electroretinogram (PERG) revealed reduced in amplitudes and delayed at the positives 100 ms (P100) and pattern visual evoked potential (PVEP) showed missing negativities at approximately 95 ms (N95) for both eyes

In ophthalmic clinic, the patient claimed to have poor vision prior to the brain aneurysm, with a best corrected visual acuity of 20/60 ever since she was in elementary school. She also reported a central scotoma in her visual field. None of the patient’s parents nor related family members showed similar symptoms. Upon further ophthalmological examinations, the dilated fundus exam showed no abnormal pigmentation on the retina and macula. However, temporal optic nerve pallor was revealed (Fig. 2A). The spectral-domain optical coherence tomography (SD-OCT) revealed a normal retina structure, with thinning of the retina nerve fiber layer around the optic nerve head, depicting optic atrophy (Fig. 2B). The full field electroretinogram (ERG) test results were within normal range, however the pattern electroretinogram (PERG) and pattern visual evoked potential (PVEP) results were not. The PERG results have missing negativities at approximately 95 ms (N95) for both eyes. For the PVEP, the positives at 100 ms (P100) were reduced in amplitudes and delayed (Fig. 3). The results from the electrophysiological test (Fig. 3) supported the findings in the retinal images (Fig. 2), which indicated optic nerve atrophy.

#### Informed consent

**Fig. 4:**
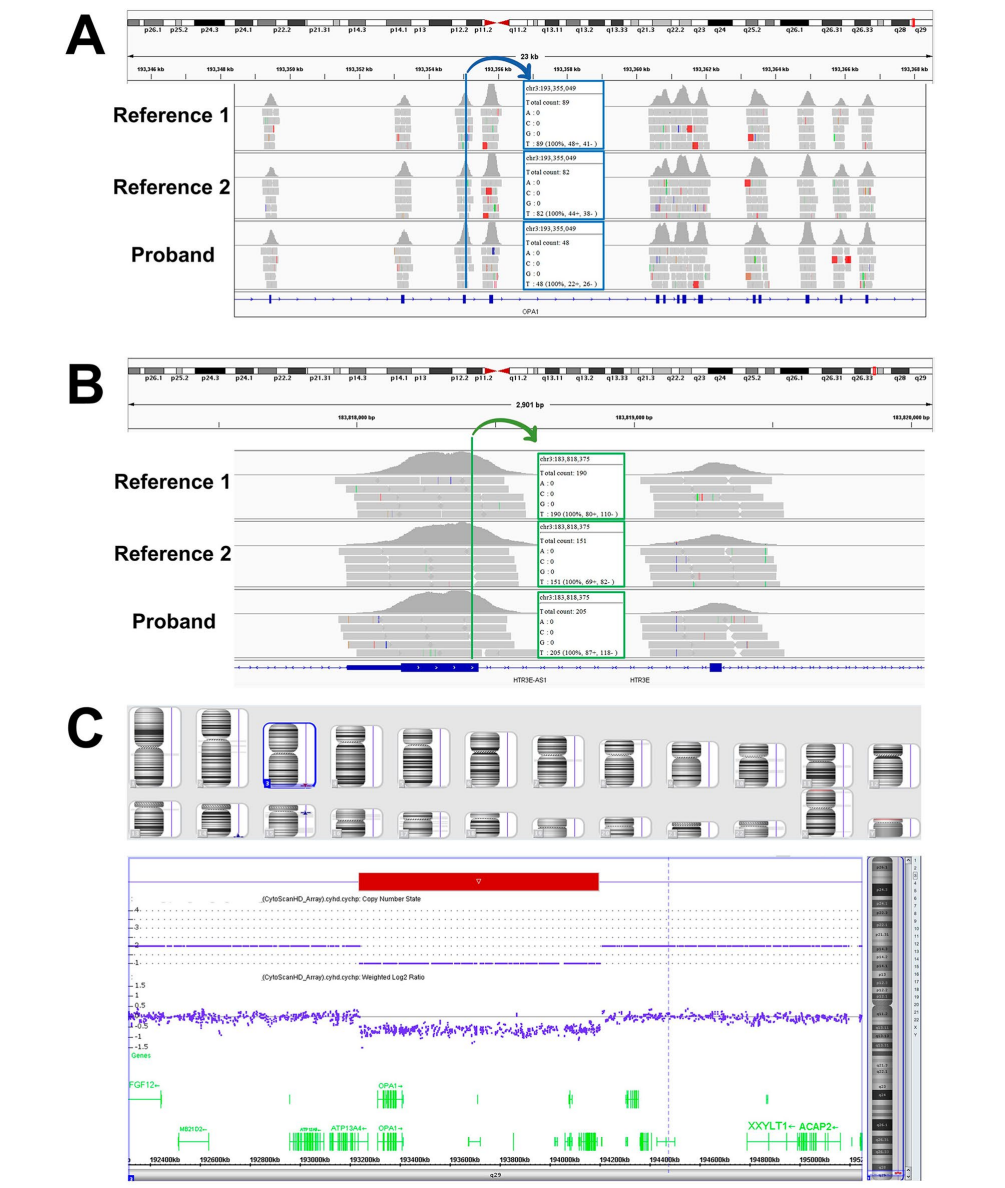
Exome sequencing and Chromosomal microarray analysis. **(A)** Approximately 50% fewer reads were observed in the *OPA1* gene in the proband’s sample compared to two other unrelated reference samples. **(B)** The reads in the neighboring genes showed similar patterns between the proband and the two unrelated reference samples. **(C)** Chromosomal microarray analysis showed a deletion of 960 kb on chromosome 3q29

for genetic testing was obtained from the patient and both parents, and the study was conducted according to the declaration of Helsinki and approved by the Institutional Review Board of Chang Gung Memorial Hospital (No. 201601569B0C602). The patient’s DNA was extracted from peripheral blood. Exome capture was performed using xGen Exome Research Panel v2 (Integrated DNA Technologies, Coralville, Iowa, USA) and sequencing was performed using NovaSeq 6000 (Illumina, San Diego, CA, USA). In total, 9,162,817,743 bases of sequence were generated and uniquely aligned to the Genome Reference Consortium Human Build 37 (GRCh37) and Revised Cambridge Reference Sequence (rCRS) of the mitochondrial genome, generating 141.19 mean depth-of-coverage within the 34,366,188 bases of the captured region, which is approximately 99.3% of the RefSeq protein coding region. Approximately 98.80% of the targeted bases were covered to a depth of ≥ 20x. In total, 67,480 single nucleotide variants (SNV) and 11,123 small insertions and deletions (indel) were identified. Due to limitations in the coverage and depth of mtDNA using exome sequencing, additional Sanger sequencing was conducted to confirm the absence of three hotspots (m.11778G > A, m.14484T > C, or m.3460G > A) associated with Leber Hereditary Optic Neuropathy in our patient. The Exome Sequencing results did not identify any disease-causing variants related to optic atrophy. Through further pipeline analysis, a deletion spanning the *OPA1* gene was suspected. Approximately 50% fewer reads were observed in the *OPA1* gene in the proband’s sample compared to two other unrelated reference samples (Fig. 4A). Meanwhile, the reads in the neighboring genes showed similar patterns between the proband and the two unrelated reference samples (Fig. 4B). Chromosomal microarray analysis (CMA) was performed with the CytoScan HD array (Affymetrix, Santa Clara, CA) according to the manufacturer’s instructions on the GeneChip™ Scanner 3000 7G platform (ThermoFisher Scientific, Waltham, US), which contains more than 2.67 million markers for copy number analysis, including 750,000 SNPs and 1.9 million nonpolymorphic probes. CMA data were visualized and analyzed with Chromosome Analysis Suite (ChAS) software package (Affymetrix, Santa Clara, CA). The results showed a 960 kb deletion on chromosome 3q29 (arr[GRCh37] 3q29(193,238,393_194,198,653)x1, Fig. 4C). This large heterozygous deletion encompasses 7 RefSeq coding-genes, namely *ATP13A4, OPA1, HES1, CPN2, LRRC15, GP5*, and *ATP13A3* genes. The Autosomal Dominant Optic Atrophy diagnosis has been confirmed by the *OPA1* heterozygous deletion identification. We have examined the asymptomatic parents who showed no signs of ocular abnormalities (as indicated in Supplement Fig. 1). Parental analysis by CMA concluded that the deletion was *de novo*.

## Discussion and conclusions

Currently, the majority of *OPA1* disease-causing variants are single nucleotide variants in exon, and *OPA1* large deletions are not commonly found [5]. To further investigate the molecular etiology, sequencing technologies (including next generation sequencing and Sanger sequencing) are commonly performed for individuals with ADOA. Fuhrmann et al. has found that genomic rearrangements in *OPA1* are frequent in patients with autosomal dominant optic atrophy (8/42), and therefore the frequency of *OPA1* copy-number variations may have been underestimated due to the sequencing technology limitations [6]. Clinical geneticists working in this field should be familiar with the mutational spectrum of *OPA1* and may use alternative methods to detect possible *OPA1* copy-number variations, such as multiplex ligation-dependent probe amplification (MLPA) and microarray [7]. Large deletions encompassing multiple genes often result in more extensive and severe phenotypes. This phenomenon is well exemplified by deletions involving the *NF1* and *RB1* genes [8, 9]. Individuals with the *NF1* deletion often develop a severe form of the disease with frequent cognitive impairment and an increased risk of tumors, in addition to typical NF1 characteristics. Likewise, individuals with *RB1* deletion often have developmental abnormalities and facial dysmorphisms, in addition to typical retinoblastoma features. In that regard, it’s important for clinicians to remain attentive to “additional phenotypes” that might manifest in patients with optic atrophy, including conditions like aneurysms. In such cases, the utilization of microarray testing could be instrumental in detecting large *OPA1* deletions that involve neighboring genes.

We have identified a 0.96 Mb deletion centromeric to the established 3q29 deletion syndrome [10]. While this deletion does not directly overlap with the well-known 3q29 deletion, it is noteworthy that it aligns with a 1.36 Mb deletion previously reported by Biamino et al. [11]. In their study, this deletion exhibited features including optic atrophy, autism, intellectual disability, psychiatric disorders, and obesity. The intriguing alignment of our findings with those reported by Biamino et al. adds a layer of complexity to our understanding of genetic variations in this region. While our patient and those in Biamino’s study both experienced optic atrophy due to the deletion of the *OPA1* gene, a notable contrast arises as our patient did not exhibit symptoms or signs related to neuropsychiatric disorders or obesity. This discrepancy may be attributed to the larger deletion in Biamino’s cases, encompassing three additional genes—*TMEM44, FAM34A*, and *LSG1*—compared to our patient’s deletion. Furthermore, the absence of brain aneurysms in the cases described by Biamino et al. suggests that this phenotype may not be directly associated with the deletion.

As mentioned previously, the patient presented in this case suffered from a brain aneurysm as well as optic atrophy, two seemingly unrelated conditions that happened coincidentally. Subarachnoid hemorrhages, life-threatening conditions resulting from blood accumulation between the arachnoid and pia mater [12], have a global incidence of 7.9 per 100,000 person-years, with women having a higher risk ratio of 1.3 [13]. This trend is mirrored in the U.S., where the incidence was 11.4 per 100,000 person-years between 2007 and 2017, with a higher incidence in women [14]. Around the world, Japan and Finland report higher cases of subarachnoid hemorrhage [13]. The incidence of SAH increases with age worldwide, particularly in women over 55 years [13]. In the U.S., individuals aged 65 or older had over five times the incidence compared to those aged 20–44 years, with women consistently having a higher incidence in each age group [14]. A study by de Rooij NK et al. further supports these findings, showing a higher incidence of SAH in men than women in the 25–45 years age group, but a reversal of this trend in the 55–85 years age group [15]. According to the microarray results, the 960 kb deletion encompasses not only the *OPA1* gene, but also neighboring genes, including *ATP13A4, HES1, CPN2, GP5, LRRC15*, and *ATP13A3*. It has been observed that among the 29 individuals reported in Decipher with deletions that overlap the 3q29 deletion identified in our study, none of them displayed brain aneurysms. This observation supports the idea that the occurrence of brain aneurysms is not directly associated with the identified deletion. However, it is important to acknowledge that the possibility of a somatic two-hit event remains a valid hypothesis. We regret that biopsy tissue testing was not conducted to explore this hypothesis further. It is plausible that the brain aneurysm phenotype found in our case is coincidental; nonetheless, we discovered that two genes, *HES1* and *CPN2*, are associated with vascular function.

Carboxypeptidase N2 (CPN2) is a type of plasma metalloprotease that has the ability to cleave basic amino acids from the C-terminal of peptides and proteins. Its primary function involves regulating vasoactive peptide hormones, growth factors, and cytokines by specifically cleaving their C-terminal basic residues [16]. Vasoactive peptide hormones are crucial signaling molecules that play a pivotal role in regulating blood vessel tone and blood pressure. They also influence the contraction or relaxation of smooth muscle cells in blood vessels, playing an essential role in maintaining hemodynamic stability and cardiovascular homeostasis. While there is currently no direct association between the function of *CPN2* and aneurysms, any dysregulation of vasoactive, any dysregulation of vasoactive peptide hormones may contribute to conditions such as vascular diseases.

HES1 belongs to a family of basic helix-loop-helix (bHLH) proteins essential for various biological processes, including neurogenesis, myogenesis, hematopoiesis, and sex determination. It serves as a transcriptional repressor for numerous genes but can also function as a transcriptional activator [17]. In the developing mouse embryo, using in situ hybridization, *Hes1* was found to be expressed in almost all endothelial cells (ECs) of the developing internal carotid artery at embryonic day 10.5 (E10.5). Notably, in almost half of *Hes1* knockout embryos, hemorrhage was observed in the cerebrospinal nervous system, indicating that Hes1 plays a role in regulating vascular remodeling and arterial fate specification of endothelial cells during brain development. Furthermore, Hes1 is identified as a critical transducer of Notch signals in brain vascular development [18]. Further functional study using a mouse model to knock out *HES1* gene could be helpful in elucidating the role of HES1 in aneurysm formation.

## Conclusion

In summary, we present a unique case featuring a *de novo* 960 kb deletion on chromosome 3q29 in a patient exhibiting optic atrophy and a brain aneurysm. The optic atrophy is conclusively attributed to the *OPA1* deletion, and the aneurysm could be a coincidental association. This case highlights the difficulties faced in interpreting CNV in syndromic cases. Clinical geneticists who specialize in this area should have knowledge of the mutational spectrum of *OPA1* and may consider using alternative techniques, such as MLPA and microarray analysis, to identify possible *OPA1* CNVs.

### Electronic supplementary material

Below is the link to the electronic supplementary material.

Supplementary Material 1 Supplementary Material 1

Supplementary Material 2

## Data Availability

The data that support the findings of this study are available on request from the corresponding author. The data are not publicly available due to privacy or ethical restrictions.
