# Supplementary figures and images for: Genetic underpinnings explored: OPA1 deletion and complex phenotypes on chromosome 3q29
Source: BMC Med Genomics. 2024 Apr 19;17:94. doi: 10.1186/s12920-024-01850-6 (PMC11031983; doi:10.1186/s12920-024-01850-6)

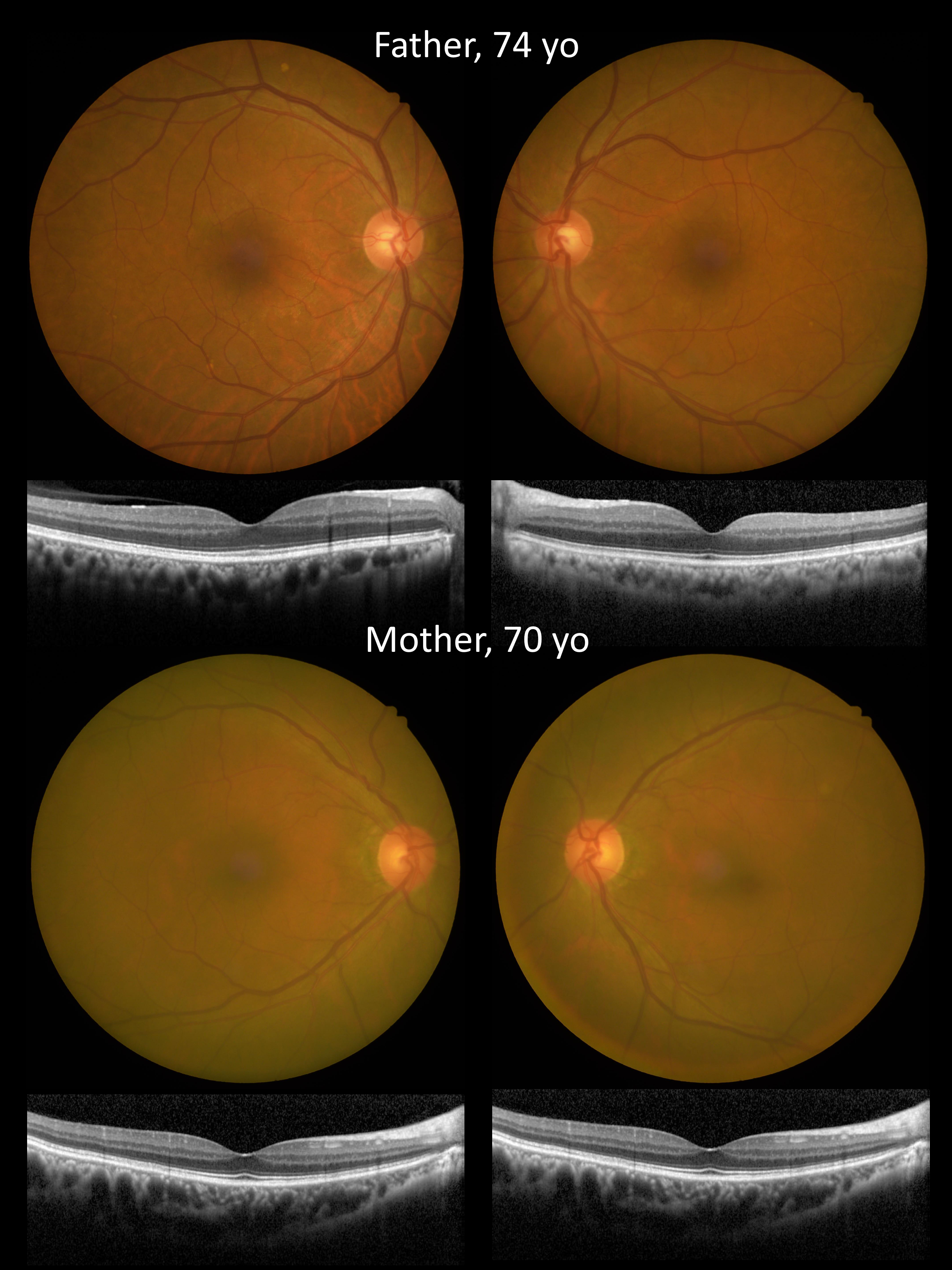

Supplement: Supplementary file 1 — Supplementary Material 1 [file 12920_2024_1850_MOESM1_ESM.png]
