# Supplementary material for: Genetic underpinnings explored: OPA1 deletion and complex phenotypes on chromosome 3q29
Source: BMC Med Genomics. 2024 Apr 19;17:94. doi: 10.1186/s12920-024-01850-6 (PMC11031983; doi:10.1186/s12920-024-01850-6)
Supplement: Supplementary file 2 — Supplementary Material 2 [file 12920_2024_1850_MOESM2_ESM.docx]

**Supplement Figure 1 Legend**

The fundus photo and spectral-domain optical coherence tomography showed a normal retina structure in the proband's father (**upper images**) and the proband's mother (**lower images**).
